# Implementing virtual reality for emergency training in nephrology: a large-scale study on educational impact and acceptance

**DOI:** 10.1093/ckj/sfag060

**Published:** 2026-02-20

**Authors:** Philipp Russ, Jonas Einloft, Muriel L Morgenschweis, Simon Bedenbender, Joy Giesen, Hendrik L Meyer, Lukas D Rimpl, Martin C Hirsch, Andre Ganser, Joseph V Bonventre, Ivica Grgic

**Affiliations:** Department of Internal Medicine and Nephrology, Marburg University, University Hospital Giessen and Marburg, Marburg, Germany; Institute for Artificial Intelligence in Medicine, Marburg University, Marburg, Germany; Department of Internal Medicine and Nephrology, Marburg University, University Hospital Giessen and Marburg, Marburg, Germany; Department of Internal Medicine and Nephrology, Marburg University, University Hospital Giessen and Marburg, Marburg, Germany; Department of Internal Medicine and Nephrology, Marburg University, University Hospital Giessen and Marburg, Marburg, Germany; Department of Internal Medicine and Nephrology, Marburg University, University Hospital Giessen and Marburg, Marburg, Germany; Department of Internal Medicine and Nephrology, Marburg University, University Hospital Giessen and Marburg, Marburg, Germany; Department of Internal Medicine and Nephrology, Marburg University, University Hospital Giessen and Marburg, Marburg, Germany; Institute for Artificial Intelligence in Medicine, Marburg University, Marburg, Germany; Department of Internal Medicine and Nephrology, Marburg University, University Hospital Giessen and Marburg, Marburg, Germany; Division of Renal Medicine, Department of Medicine, Brigham and Women’s Hospital, Harvard Medical School, Boston, MA, USA; Department of Internal Medicine and Nephrology, Marburg University, University Hospital Giessen and Marburg, Marburg, Germany; Institute for Artificial Intelligence in Medicine, Marburg University, Marburg, Germany

**Keywords:** acute kidney injury (AKI), anti-neutrophil cytoplasmic antibody–associated vasculitis, nephrology emergency medicine, rapidly progressive glomerulonephritis (RPGN), virtual reality (VR)

## Abstract

**Background:**

Rapidly progressive glomerulonephritis (RPGN) is a prototypical nephrology emergency requiring rapid recognition and treatment. Despite the promise of immersive virtual reality (VR), nephrology lacks VR-based emergency training. Building on our initial VR simulation, we evaluated the feasibility, acceptance and effectiveness of large-scale curricular implementation.

**Methods:**

On the STEP-VR platform, we embedded a custom three-dimensional RPGN case as a mandatory internal medicine module for fifth-year students. Knowledge was measured pre- and post-training. Instructional design effects were tested by comparing tutor-guided versus tutorless sessions and by examining a pretesting effect. Acceptance and simulation sickness were surveyed.

**Results:**

A total of 408 students participated (mean age 25.4 years, 59.6% female). In the pretest–posttest cohort, knowledge increased after training (overall relative gain +101%; *P* < .001, Cohen’s *d* = 1.30). After adjustment, tutor-guided sessions yielded higher posttest scores than tutorless sessions (+5.5 points on a 0–49 scale). Between-cohort posttest comparisons suggested an exploratory forward-testing effect (*P* ≤ .01). Improvements spanned RPGN recognition, urine sediment examination, laboratory and histology interpretation and hyperkalaemia management. Acceptance was high and simulation sickness was infrequent.

**Conclusions:**

This study demonstrates the successful large-scale curricular integration of VR-based training for nephrology emergencies, confirming its feasibility and educational effectiveness. By sustainably embedding immersive VR into medical education, this approach bridges a long-standing training gap in nephrology, enhances clinical competencies, increases student engagement with the specialty and may contribute to long-term efforts to address the nephrology workforce shortage.

## Graphical Abstract

**Graphical Abstract:**
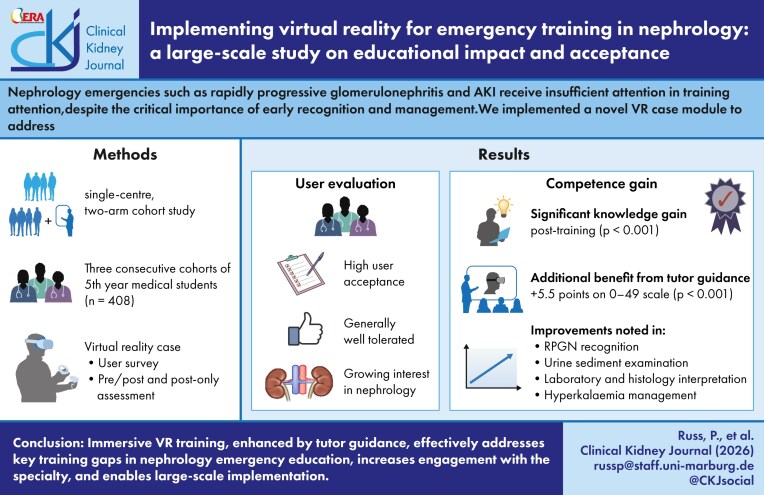

KEY LEARNING POINTS **What was known:**

- Immersive virtual reality (VR) improves emergency training in several specialties, but nephrology lacked curriculum-embedded VR modules and robust evidence on large-scale feasibility, acceptance and learning outcomes.
- Rapidly progressive glomerulonephritis (RPGN) requires rapid recognition and treatment to prevent irreversible kidney damage; many students report low preparedness for emergencies and nephrology is often underrepresented in undergraduate curricula.
- Evidence on instructional design factors, including synchronous tutoring and pretesting, in VR-based nephrology education was limited and prior work comprised small-scale prototypes without curriculum-wide implementation.

**This study adds:**

- Cohort-scale curricular implementation across three consecutive semesters (*N* = 408) demonstrated high acceptance, infrequent simulation sickness and operational feasibility at the cohort scale.
- In a pretest–posttest cohort, knowledge increased after VR training, tutor guidance improved posttest performance and remained beneficial after adjustment and between-cohort posttest comparisons suggested an exploratory pretesting advantage.
- Item-level performance improved across key domains, including RPGN recognition, urine sediment examination, interpretation of laboratory and histology findings and hyperkalaemia management.

**Potential impact:**

- This approach provides a reproducible, resource-efficient blueprint for embedding immersive nephrology emergency training in medical curricula to improve preparedness for acute kidney injury complications and enable earlier recognition and treatment of RPGN.
- Curriculum integration can increase the visibility and appeal of nephrology to students and may help support recruitment into the specialty.
- Where feasible, programme delivery should prioritize tutor-supported sessions and include brief pretests to maximize learning gains for a given educational resource.

## INTRODUCTION

Preparedness for acute clinical care remains an ongoing challenge in medical education. Medical students and junior residents frequently report limited readiness to manage these critical situations in practice [1–3]. The primary reason cited for this gap is a lack of practical experience, which can limit their ability to confidently navigate complex scenarios [4, 5]. Immersive technologies, such as virtual reality (VR), have emerged as a simulation modality that can provide interactive, standardized scenarios in a safe learning environment [6, 7]. By requiring learners to actively gather information and make management decisions, VR-based training may support competence development and strengthen confidence in acute care contexts [8, 9]. Across medical education settings, acceptance of VR is commonly reported to be high and learners often advocate for broader curricular integration of such innovative training methods [10, 11].

Beyond limited preparedness, nephrology is often perceived as highly complex, and negative attitudes and low confidence among learners have been described as ‘nephrophobia’ [12]. In a European survey, 64% of respondents reported that medical students view nephrology unfavourably and consider it a less attractive career option; limited undergraduate contact and perceived complexity were key deterrents, whereas role models and early practical experience supported interest [13]. Similarly, a national survey among medical students and internal medicine residents identified subject exposure and access to mentors as important factors shaping career considerations [14].

VR-based emergency training has been evaluated in several domains (e.g. trauma care, resuscitation and cardiology), with evidence of improved preparedness and skills in simulated settings [9, 15, 16]. In nephrology, however, immersive emergency training remains scarce, even though nephrology emergencies are increasingly recognized as time-critical conditions in routine care. Clinically, delayed recognition of acute nephrology conditions can contribute to long-term kidney sequelae, including chronic kidney disease (CKD), a major and costly public health burden [17, 18]. Early recognition of nephrology emergencies and timely escalation of diagnostic and specialist care can shorten the time to therapy and thereby improve renal and patient outcomes [19]. For example, untreated RPGN has been associated with a 1-year mortality rate of 80%, whereas timely treatment can improve the prognosis to a 5-year survival rate of 75% [20]. Moreover, high-risk acute kidney injury (AKI) complications, such as severe hyperkalaemia, are clinically important and underscore the need for practical training in their management [21, 22].

Building on our previously reported development of a comprehensive VR-based nephrology emergency training module [23], we prepared the optimized RPGN scenario for large-scale curricular implementation. The module is centred on anti-neutrophil cytoplasmic antibody (ANCA)-associated vasculitis, the most common cause of RPGN [24], and reflects nephrology-specific clinical presentations, diagnostic workflows and therapeutic interventions.

The aim of the present study was to evaluate large-scale curricular implementation of the optimized scenario across entire student cohorts and assess predefined learning objectives for managing nephrology emergencies. Moreover, we examined the impact of different instructional designs on knowledge gain and assessed student acceptance and tolerability, including simulation sickness.

## MATERIALS AND METHODS

### Participants and study design

To evaluate the impact of VR-based training on student competence, we conducted a single-centre study with senior fifth-year medical students at Marburg University. The study period spanned the summer semester 2023 (cohort A, pretest only), winter semester 2023–2024 (cohort B, pretest and posttest) and summer semester 2024 (cohort C, posttest only). The training was integrated into the curricular internal medicine internship. Development of the beta version and the initial pilot implementation have been reported previously [23]. That report included cohort A (120/134) and a subset of cohort B (81/160) participants. In the present article, cohort A is used only to characterize baseline prior knowledge. The primary contribution is the scaled curricular rollout across three consecutive semesters, with expanded analyses and outcomes, including comparisons of tutor-guided versus tutorless instruction, an exploratory pretesting effect, cluster-adjusted analyses and evaluation of acceptance and simulation sickness.

In the summer semester 2023, during the beta implementation (for a detailed description, see the development study) [23], students completed a baseline prior-knowledge assessment, forming cohort A (pretest only). Cohort A participated in small peer groups of four to eight students and subsequently provided case evaluation feedback; by design, no posttest was administered. For cohort B (winter semester 2023–2024; pretest and posttest), students first completed the same baseline prior-knowledge assessment (pretest, before VR exposure) followed by a brief orientation. The VR-based emergency training was then conducted as a peer group seminar in groups of four to six students, either with or without synchronous guidance from a trained student tutor. Groups were allocated 1:1 to the two seminar modes using an alternating sequence across consecutive sessions to ensure a balanced distribution across the 8-week teaching period: tutor-guided mode in which the tutor provided technical assistance, moderated the seminar and offered medical guidance; and tutorless mode, in which the tutor was available only for technical issues. Following training, a post-training questionnaire was used to assess the learning outcomes. To minimize potential bias from feedback on the pretest, correct pretest answers were not disclosed.

**Figure 1:**
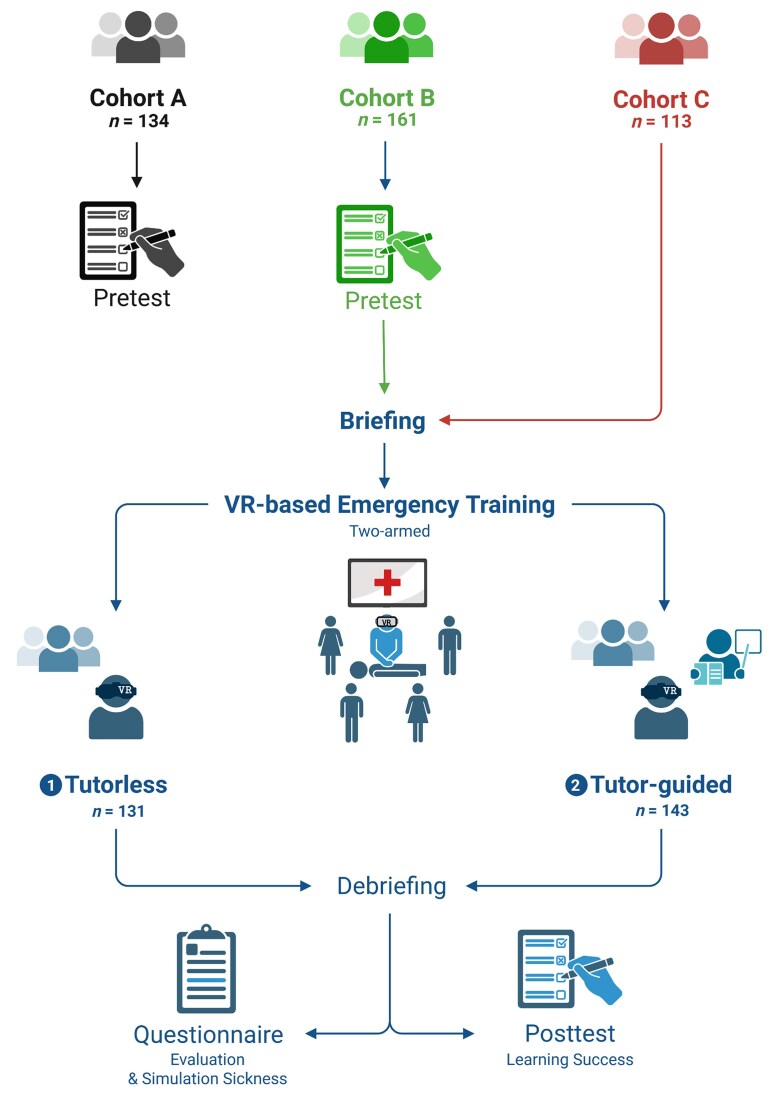
Study flow chart. Three cohorts were included. Cohort A (pretest only), cohort B (pretest + posttest) and cohort C (posttest only). After a technical briefing, participants in cohorts B and C were allocated 1:1 to either a tutor-guided or tutorless seminar mode using an alternating sequence across consecutive sessions. Post-training learning outcomes and acceptance/tolerance were assessed using a knowledge test and questionnaires. Created in BioRender (Bedenbender, S., 2025; https://BioRender.com/zoibq2h).

To explore potential test sensitization (a forward-testing effect), cohort C (summer semester 2024) was designed as a posttest-only cohort and did not complete a pretest before VR exposure. This design enabled exploratory between-cohort comparisons of posttest scores (cohort B versus cohort C), stratified by seminar mode. Students were again divided into peer groups of four to six students and allocated 1:1, using the same alternating sequence as the tutor-guided versus tutorless sessions across the 8-week teaching period. After completing the VR-based training, learning outcomes were evaluated using the same questionnaire. In addition, students in cohort B and cohort C completed an evaluation survey and a simulation sickness questionnaire (Fig. 1). Completion of evaluation questionnaires and use of data were voluntary. Throughout the article, ‘baseline prior knowledge’ refers to pretest performance before VR exposure among students who completed a pretest (cohorts A and B).

### VR application

We used commercially available Meta Quest 2 (Meta Platforms, Menlo Park, CA, USA) head-mounted displays (HMDs). These VR glasses are portable, support inside-out tracking and enable wireless connection to the host computing device, allowing freedom of movement. The host device was an Acer Predator Helios 300 laptop equipped with an Intel Core i7-10750H CPU, NVIDIA GeForce RTX 3080 Laptop GPU with 8 GB VRAM and 32 GB DDR5 RAM. Wireless streaming to the VR-HMD was facilitated via Meta Quest Air Link, using an Asus AX5400 router. For the three-dimensional and interactive RPGN simulation, we utilised the STEP-VR platform (short for Simulation-based Training of Emergencies for Physicians using Virtual Reality), originally developed by ThreeDee (Munich, Germany) and now operated by OrangeWhip Interactive UG (Germering, Germany) [25, 26]. STEP-VR, originally developed in collaboration with the medical faculty of the University of Würzburg, includes several emergency scenarios [27]. Our nephrology emergency module, created in collaboration with ThreeDee, builds on this platform [23].

### Simulation design

**Figure 2:**
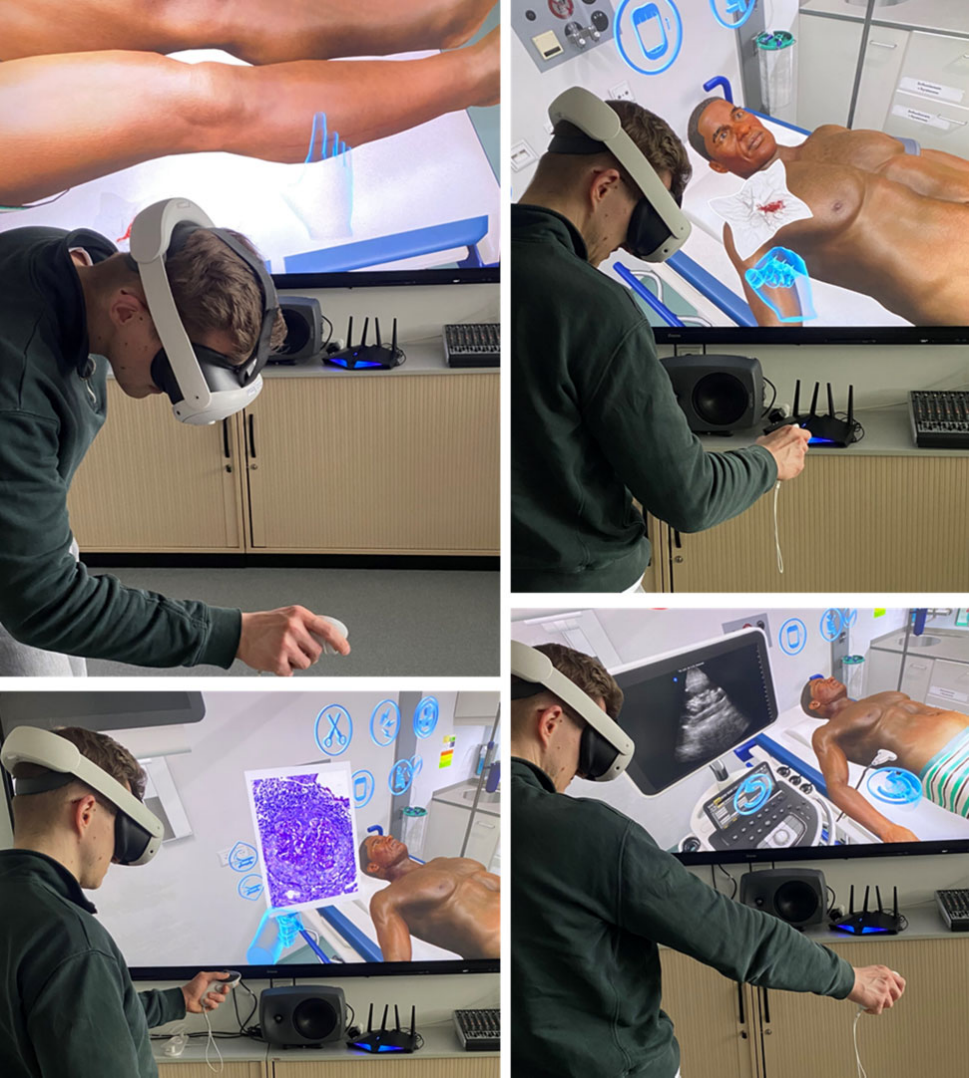
Snapshots illustrating the immersive, interactive VR training as trainees manage a realistic nephrology emergency case. The simulation includes medical history taking, patient inspection, physical examination and a range of diagnostic and therapeutic options. Trainees are fully immersed in a three-dimensional virtual emergency room with full freedom of movement, providing a realistic clinical environment. QR codes for short demonstration videos are provided in Supplementary Fig. 1.

Following a briefing session, which included an introduction to the HMD and software, one student actively went through the case while wearing the VR glasses. Meanwhile, the other students observed the scenario and provided ideas and advice to the ‘active player’ on diagnostic and therapeutic procedures. As outlined in our development report [23], the virtual case involves a middle-aged patient who presents with dizziness, weakness, leg oedema, bloody cough and reduced urine output. The scenario requires students to take a detailed history (implemented via predefined, author-scripted dialogue options, accessed through interactive icons), perform a physical examination and utilise appropriate diagnostic tools such as electrocardiogram (ECG), X-ray, computed tomography scan, ultrasound, detailed laboratory and urinalysis (including urine dipstick and sediment) and ultimately a renal biopsy. Students must also manage life-threatening hyperkalaemia as a complication of AKI, recognize underlying RPGN and initiate its appropriate treatment. At the conclusion of the simulation, an internal program generates feedback, assessing performance based on the percentage of key medical objectives achieved. An impression of the virtual case is shown in Fig. 2, a summary of the case is presented in Supplementary Table 1 and QR codes for short demonstration videos are provided in Supplementary Fig. 1.

### Questionnaire design

The knowledge and learning assessment questionnaire consisted of 17 open-ended questions, each with different weightings related to the clinical presentation, diagnosis and treatment of RPGN and the complications of AKI in general. The maximum achievable score was 49 (see Supplementary Table 2). Additionally, the evaluation sheet included 16 scaled questions and 2 open-ended questions, while the simulation sickness questionnaire contained 16 questions assessed on a 4-point Likert scale (Supplementary Table 3) [28].

### Data analysis

The survey data were collected from printed questionnaires completed by the students. All responses to the questions were digitized into an Excel spreadsheet (version 16; Microsoft, Redmond, WA, USA), which served as the basis for analysis. Data were analysed and graphs plotted using Excel. Both the flow chart and, in part, the graphical abstract were created using BioRender (https://www.biorender.com/; BioRender, Toronto, ON, Canada). Learning success [delta score (Δ)] was defined as the within-student change in the total score (posttest − pretest) and was therefore only calculated in cohorts with paired measurements (cohort B). In cohort C (no pretest by design), individual delta scores could not be computed; analyses therefore focused on posttest performance and—when exploring a pretesting effect—between-cohort comparisons of posttest scores with baseline prior-knowledge performance characterized using pretest data from students who completed a pretest (cohorts A and B). For within-student comparisons in cohort B (pretest and posttest), we used paired *t*-tests and assessed the normality of the difference scores. Between-group comparisons (tutor-guided versus tutorless) and between-cohort comparisons (cohort B versus cohort C) were performed using independent two-sample *t*-tests; homogeneity of variances was assessed using the Levene test. For paired pre–post comparisons, effect sizes (Cohen’s *d*) were calculated as the standardized mean change based on the standard deviation (SD) of within-student change scores. Additionally, learning outcomes were examined at the item level descriptively (pretest versus posttest). To address potential confounding and group-level clustering, we performed exploratory multivariable analyses in R (version 4.5.0; R Foundation for Statistical Computing, Vienna, Austria). In cohort B, posttest scores were modelled as a function of seminar mode, pretest score, age and gender (self-reported); in cohort C, posttest scores were modelled as a function of seminar mode, age and gender. Because allocation occurred at the small-group level, we used cluster-robust standard errors (SEs) at the seminar-group level (35 seminar groups per cohort). As sensitivity analyses, we fitted mixed effects models with a random intercept for seminar group; interaction terms (pretest × mode, mode × age, mode × gender) were explored in cohort B. Results were defined as statistically significant when α was ≤0.05.

### Ethical approval

Permission to conduct this study was granted by the Ethics Committee of the Medical Faculty of Philipps University of Marburg (file number 23-218). This research was carried out in accordance with the Declaration of Helsinki and complied with local guidelines for human studies.

## RESULTS

### Baseline characteristics

A total of 408 students participated between the summer semester 2023 and the summer semester 2024. Among participants, 243 (59.6%) identified as female, 137 (33.6%) as male, 1 (0.2%) as non-binary and 27 (6.6%) did not specify. The mean age was 25.4 years (SD 2.7; range 20–40 years; median 25). Cohort sizes were 134 (cohort A), 161 (cohort B) and 113 (cohort C). Across cohorts and between seminar modes, baseline demographics and group size were broadly similar (Tables 1–3).

**Table 1:** Baseline characteristics by cohort.

Characteristics	Cohort A (*n* = 134)	Cohort B (*n* = 161)	Cohort C (*n* = 113)
Age (years), mean (SD)	25.1 (2.6)	25.7 (3.0)	25.5 (2.5)
Gender, *n* (%)			
Female	84 (62.7)	92 (57.1)	67 (59.3)
Male	42 (31.3)	57 (35.4)	38 (33.6)
Non-binary	1 (0.7)	0	0
Not specified	7 (5.2)	12 (7.5)	8 (7.1)
Pretest score, mean (SD)	10.4 (5.7)	9.9 (5.1)	Not assessed
Group size (students) mean (SD)	6.6 (1.1)	4.9 (0.3)	4.6 (0.5)

**Table 2:** Cohort B baseline characteristics by seminar mode.

Characteristics	Tutor-guided (*n* = 81)	Tutorless (*n* = 80)
Age (years), mean (SD)	26.1 (3.2)	25.1 (2.8)
Gender, *n* (%)		
Female	44 (54.3)	48 (60)
Male	33 (40.7)	24 (30)
Non-binary	0	0
Not specified	4 (4.9)	8 (10)
Pretest score, mean (SD)	9.7 (5.0)	10.2 (5.2)
Group size (students), mean (SD)	4.5 (0.8)	5 (0.6)

**Table 3:** Cohort C baseline characteristics by seminar mode.

Characteristics	Tutor-guided (*n* = 62)	Tutorless (*n* = 51)
Age (years), mean (SD)	25.5 (2.4)	25.4 (2.7)
Gender, *n* (%)		
Female	34 (54.8)	33 (64.7)
Male	24 (38.7)	14 (27.5)
Non-binary	0	0
Not specified	4 (7.8)	4 (7.8)
Pretest score	Not assessed	Not assessed
Group size (students), mean (SD)	4.5 (0.5)	4.6 (0.5)

### Knowledge gain through VR and additional benefit of tutor guidance

**Figure 3:**
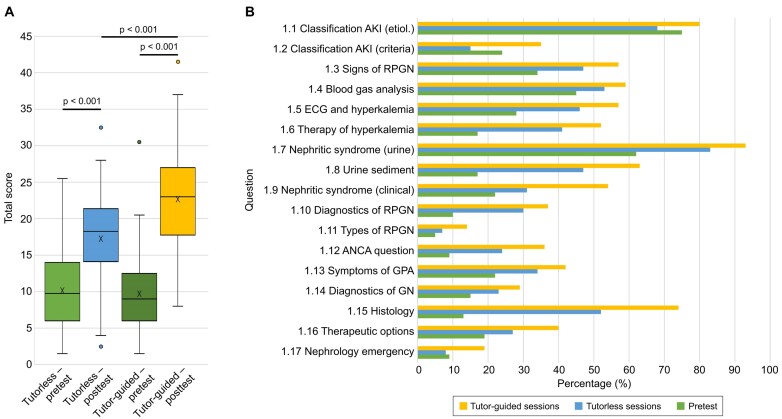
Knowledge gain through VR-based training. **(A)** Cohort B (pretest + posttest). Box plots show the median (line), mean (X) and interquartile range (box). Paired tests were used for within-student pre–post comparisons and unpaired tests for between-mode posttest comparisons. Pre–post gains were significant in both seminar modes (*P* < .001). Posttest scores were higher with tutor guidance (*P* < .001). **(B)** Item-level performance shown descriptively. Bars indicate the percentage of correct responses per item for baseline prior knowledge (pooled pretest scores from cohorts A + B) and post-training performance (pooled posttest scores from cohorts B + C), stratified by seminar mode (tutorless versus tutor-guided).

In cohort B (pretest and posttest), baseline prior knowledge was comparable between seminar modes. Across cohort B overall (*n* = 161), mean scores increased from 9.9/49 (SD 5.1) to 20.0/49 (SD 6.8) (*P* < .001; Cohen’s *d* = 1.30), corresponding to a relative gain of 101%. In tutorless sessions (*n* = 80), mean pretest scores were 10.2/49 (SD 5.2) and increased to 17.3/49 (SD 5.9) post-training (*P* < .001; Cohen’s *d* = 1.09). With tutor guidance (*n* = 81), mean pretest scores were 9.7/49 (SD 5.0) and increased to 22.7/49 (SD 6.7) post-training (*P* < .001; Cohen’s *d* = 1.66). Posttest performance was significantly higher in tutor-guided than in tutorless sessions (*P* < .001) (Fig. 3A).

On an item-level basis, participants demonstrated improvements across most domains post-training, including recognition of RPGN and key clinical features, urine diagnostics in suspected RPGN (dipstick and sediment), interpretation of laboratory and histology findings and hyperkalaemia management. Fig. 3B displays item-level performance descriptively using pooled baseline prior-knowledge data (pretest, cohorts A + B) and pooled post-training performance (posttest, cohorts B + C), stratified by seminar mode. The full questionnaire is available in Supplementary Table 2.

To address potential confounding and group-level clustering, we performed exploratory multivariable analyses. In cohort B, tutor guidance remained associated with higher posttest scores after adjustment for pretest score, age and gender (adjusted difference: +5.5 points, SE 1.4, *P* < .001). There was no evidence of effect modification by baseline knowledge (pretest × mode interaction *P* = .403) or by age or gender (mode × age *P* = .882; mode × gender *P* = .285). In a mixed effects sensitivity analysis accounting for clustering at the seminar-group level (random intercept for the seminar group), results were consistent (+5.4 points, *P* < .001). In cohort C, adjusted analyses similarly supported the benefit of tutor guidance (+5.9 points, *P* = .0037).

### Pretesting effect

Because cohort C did not complete a pretest by design, within-student delta scores (Δ = posttest − pretest) could only be calculated for cohort B (pretest and posttest). To explore a potential forward-testing effect we compared posttest performance in cohort B (pretest and posttest) versus cohort C (posttest only), stratified by seminar mode. Baseline prior knowledge corresponds to the pretest before VR exposure among students who completed a pretest (cohorts A and B).

In cohort C, mean posttest scores were 14.2 points (SD 6.1) in tutorless sessions and 20.3 points (SD 5.2) in tutor-guided sessions. In cohort B, students who completed a pretest before training achieved slightly higher posttest scores: 17.3 points (SD 5.9) in tutorless sessions and 22.7 points (SD 6.7) in tutor-guided sessions.

**Figure 4:**
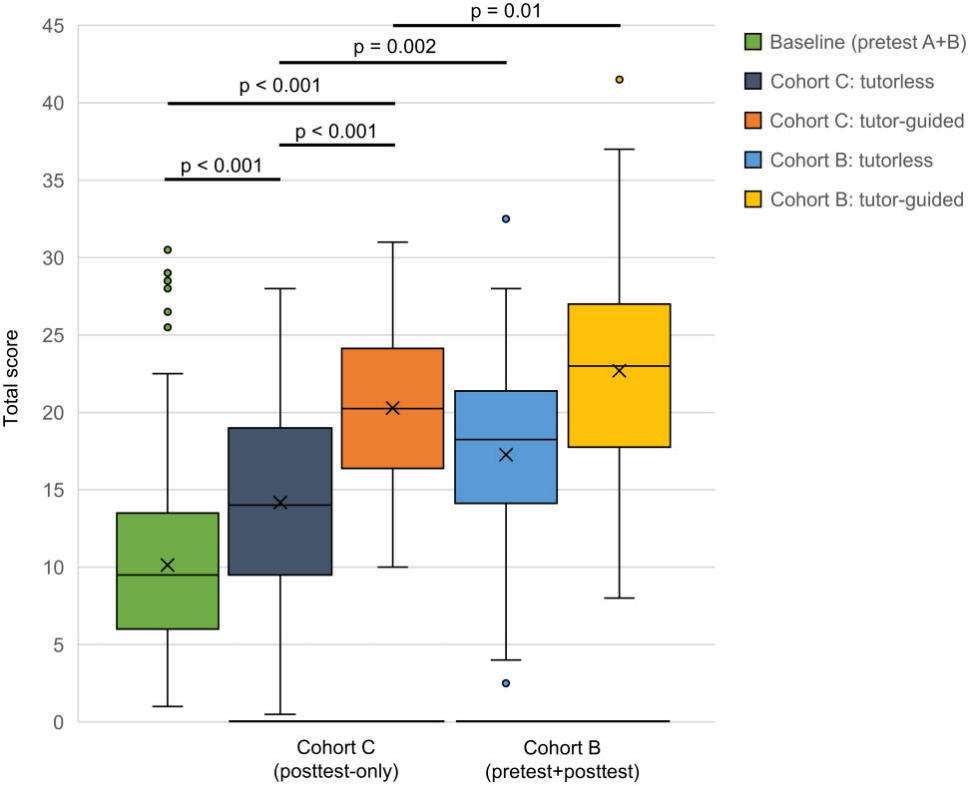
posttest performance by cohort and seminar mode (exploratory pretesting comparison). Baseline prior knowledge represents pooled pretest scores (cohorts A + B). Cohort B values reflect posttest scores. All brackets denote unpaired between-cohort comparisons. Between-cohort comparisons are exploratory, as cohorts were assessed in different semesters.

In both seminar modes, cohort C posttest scores exceeded baseline pretest performance (both *P* < .001). Between cohorts, posttest scores were higher in cohort B than in cohort C in tutorless (*P* = .002) and tutor-guided sessions (*P* = .01), consistent with an exploratory pretesting advantage (Fig. 4).

### Evaluation of acceptance and tolerance

We assessed acceptance using a numeric rating scale ranging from 1 (lowest) to 10 (highest). Overall, participants rated the VR case and emergency setting very positively. Mean ratings averaged 8.0/10 in tutor-guided sessions and 7.7/10 in tutorless sessions (*P* < .001).

**Figure 5:**
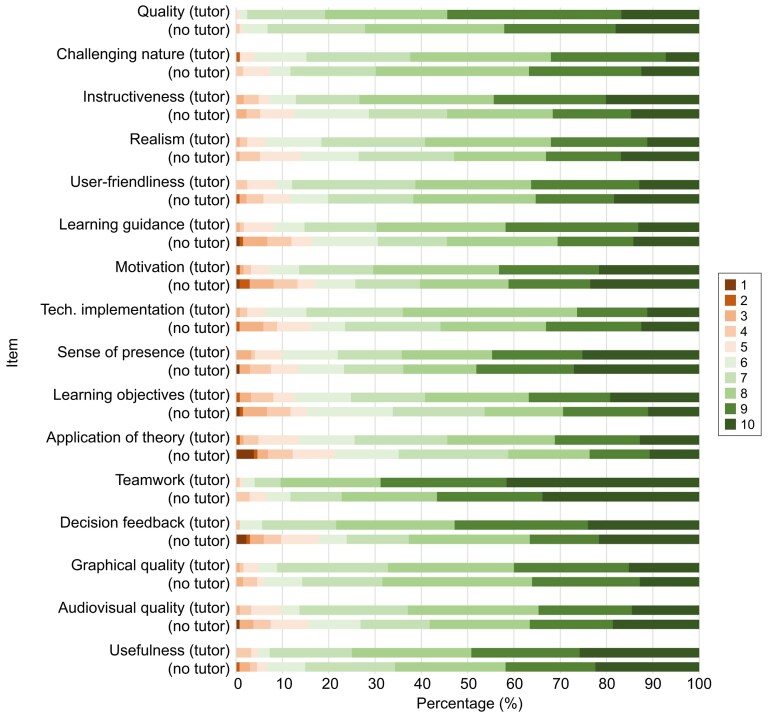
Evaluation of the virtual scenario by seminar mode. Stacked bars show the percentage distribution of ratings (1–10) for each evaluation item, shown side by side for tutor-guided and tutorless sessions. Ratings range from 1 (does not apply at all/strongly disagree) to 10 (fully applies/strongly agree). The full evaluation questionnaire is provided in Supplementary Table 3.

High ratings were observed in both modes for quality (8.5 versus 8.2; *P* = .076), realism (7.8 versus 7.6; *P* = .372) and usefulness for practice (8.4 versus 8.0; *P* = .079). Compared with tutorless sessions, tutor-guided sessions received significantly higher ratings for motivation (8.1 versus 7.6; *P* = .034) and for perceived ability to apply theoretical knowledge (7.5 versus 6.9; *P* = .013). Furthermore, tutor guidance was associated with higher perceived feedback quality (*P* < .002 and *P* < .001; see Fig. 5).

Overall, across evaluation items, tutor-guided sessions received higher ratings for motivation, perceived applicability of knowledge and perceived feedback quality. The full evaluation questionnaire is available in Supplementary Table 3.

In open-ended questions, several students reported increased interest in nephrology and described the case as engaging and challenging.

**Figure 6:**
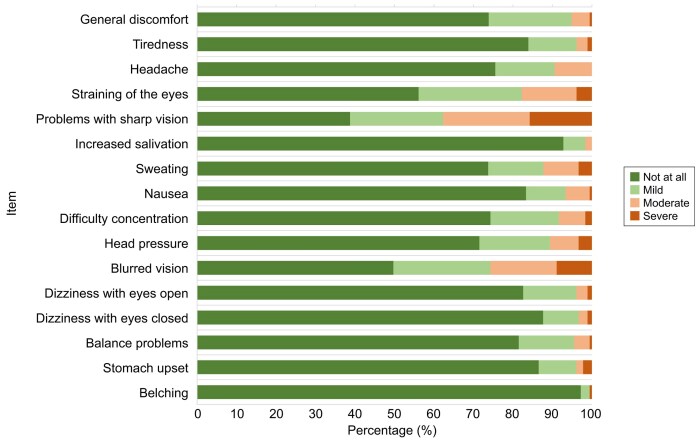
Simulation sickness. Bars show the percentage distribution of responses across the 4-point Likert scale. Overall, tolerability was high.

In general, the simulation was well tolerated. Regarding simulation sickness, most participants reported no or only mild symptoms across items. For non-visual symptoms, the proportion reporting no or only mild symptoms ranged from 87.7% to 99.4% [e.g. sweating: 157/179 (87.7%); nausea: 168/180 (93.3%); dizziness with eyes open: 171/178 (96.1%); stomach upset: 171/178 (96.1%); belching 176/177 (99.4%)]. Visual symptoms were reported more frequently. Eye strain and blurred vision were most commonly rated as none or only mild [eye strain: 148/180 (82.2%); blurred vision: 133/179 (74.3%)], whereas reduced visual sharpness was more often rated as moderate or severe [67/178 (37.6%)] (Fig. 6). Item-level distributions are provided in Supplementary Table 4.

## DISCUSSION

In this study we successfully implemented a VR-based nephrology emergency training module at the cohort scale within the medical curriculum across three consecutive semesters (*N* = 408). In the pretest–posttest cohort, knowledge increased significantly after VR training (overall relative gain 101%; *P* < .001) and tutor guidance remained associated with higher adjusted posttest scores (+5.5 points). Acceptance was high and simulation sickness was infrequent. Together, these findings support the feasibility of curricular implementation and highlight tutor support as a relevant instructional design factor that can further enhance knowledge gains in immersive VR-based nephrology education. This feasibility is particularly important because medical school often provides students with their first structured exposure to nephrology, creating an opportunity to foster engagement with this specialty [29, 30]. Through large-scale curricular integration, this programme addresses a long-standing educational gap in practice-oriented training for nephrology emergencies.

These findings align with a growing body of evidence supporting simulation-based learning as an effective educational strategy. Despite certain limitations, simulation-based learning is increasingly used in high-stakes training environments (e.g. disaster management, law enforcement) and in medical education, where it can support safety, skill acquisition and teamwork [31]. In nephrology, it enables risk-free training tailored to clinical practice, can help nephrologists maintain technical proficiency (e.g. kidney biopsy) and may support attracting younger professionals to the field [32].

Managing medical emergencies presents a distinct challenge, particularly for medical students and early-career healthcare professionals, who often feel inadequately prepared for such high-stakes situations. In nephrology, this challenge may be amplified by ‘nephrophobia’, a term used to describe negative attitudes toward nephrology and low confidence among learners in diagnosing and managing renal conditions [12]. Surveys consistently report that nephrology is perceived as particularly complex and challenging and that limited undergraduate exposure and few clinically contextual learning opportunities contribute to these perceptions and may reduce the attractiveness of the specialty [13, 14]. Against this backdrop, our high acceptance ratings as well as the open-ended feedback indicating increased interest in nephrology suggest that immersive, practice-oriented VR training can offer an engaging entry point into nephrology emergencies and may help mitigate perception-related barriers.

While integrating these programmes into mandatory curricula is crucial, logistical and funding challenges remain, requiring innovative solutions and collaboration between academia and industry. Moreover, continued research is needed to evaluate the impact of simulation-based learning on clinical outcomes.

In this context, our study provides further insights into specific factors that enhance the effectiveness of VR-based learning. Our findings highlight the greater knowledge gains achieved with tutor support, which is consistent with the prevailing literature showing that motivation and emotional encouragement from educators can substantially improve student learning [33]. Although tutor guidance requires additional personnel resources, the digital capabilities of VR offer an opportunity to optimize resource use by reducing dependence on simulated patients and medical equipment in educational settings. A previously reported amortized per-student cost estimate was US$4.50 [23]. Based on this estimate, the mean knowledge gains observed in cohort B correspond to ≈US$0.35–0.63 per point gained (tutor-guided versus tutorless). This calculation is intended as an illustrative estimate and depends on local cost structures and implementation scale.

Furthermore, students who completed the learning objectives test prior to training showed significantly better post-training performance. This pattern is consistent with established literature on test-enhanced learning and the forward effect of testing, which indicates that retrieval practice can facilitate subsequent learning and retention of new information [34–36]. Brief pretesting may therefore facilitate learning during the VR session by increasing attention and strengthening the encoding of new information, which may improve later recall [34].

Overall, the VR training was well-accepted, reinforcing its feasibility as a teaching tool. Students rated the technology highly in terms of relevance, practical utility and overall acceptance. Qualitative feedback suggested that the scenario was perceived as engaging and clinically relevant, consistent with prior reports on learner preferences for interactive simulation formats [10, 37, 38].

Despite these encouraging results, several limitations should be acknowledged. First, our study design evaluated only immediate knowledge gains following a single training session, thus long-term effects such as retention and transfer to clinical performance remain unassessed. Second, we did not directly compare VR-based training with traditional instructional formats (e.g. lecture-based or case-based teaching or human-based simulation). This limits conclusions regarding comparative effectiveness and cost-effectiveness relative to established teaching methods. Moreover, this study focused on a single, highly detailed virtual case and patient avatar. While this complexity may have enhanced immersion and diagnostic reasoning, additional scenarios covering a broader range of nephrology emergencies are needed to assess generalizability. Finally, the relatively short duration of each session and the comparatively large group sizes may have influenced learning outcomes, as opportunities for individual interaction and repetition were limited. However, we believe that, if anything, these constraints may have attenuated the observed learning gains.

Future research should focus on longitudinal evaluation of knowledge retention and transfer to clinical performance and should address implementation questions relevant to routine curricular delivery (e.g. staffing models for instructional support, fidelity across sites and scalability of tutor input through embedded guidance). Multicentre implementations and comparative studies against established teaching formats will be important to define the optimal role of VR within nephrology education.

## CONCLUSIONS

This study demonstrates successful large-scale curricular integration of VR-based training for nephrology emergencies, supporting its feasibility, scalability and educational effectiveness. Immersive, practice-oriented simulation substantially improved students’ knowledge, while achieving high acceptance and minimal adverse effects. Beyond enhancing preparedness for acute nephrology scenarios, the engaging nature of VR training fosters curiosity and may promote sustained interest in nephrology as a specialty. By embedding such innovative approaches into medical curricula, immersive VR could strengthen clinical competencies and support long-term educational transformation and could ultimately contribute to improving the quality of patient care.

Future studies should build on these results by comparing VR-based and traditional instructional methods and by assessing long-term retention and transfer to clinical performance.

## Supplementary Material

sfag060_Supplemental_File

## Data Availability

All relevant data are reported in the article. Additional data can be provided by the corresponding author upon reasonable request.
