# Supplementary material for: Implementing virtual reality for emergency training in nephrology: a large-scale study on educational impact and acceptance
Source: Clin Kidney J. 2026 Feb 20;19(5):sfag060. doi: 10.1093/ckj/sfag060 (PMC13166867; doi:10.1093/ckj/sfag060)
Supplement: sfag060_Supplemental_File [file sfag060_supplemental_file.docx]

Supplementary Material

***“Implementing Virtual Reality for Emergency Training in Nephrology: A Large-Scale Study on Educational Impact and Acceptance”***

**Table of Contents for the Supplementary Material**

| Supplementary Table 1 \| Description of the case. |
| --- |
| Supplementary Table 2 \| Design of the learning assessment questionnaire. |
| Supplementary Table 3 \| Design of the evaluation and simulation sickness questionnaire. |
| Supplementary Table 4 \| Item-level distribution of Simulation Sickness Questionnaire (SSQ) responses after VR training. |
| Supplementary Figure 1 \| QR codes of the scenario. |

Supplementary Table 1 | Description of the case.

| **Medical History and Clinical Examination** |  |  |
| --- | --- | --- |

Our case centers on a 51-year-old patient presenting with primary symptoms of dizziness and hemoptysis. The medical history unfolds step by step, allowing the user to gather specific details about the patient’s presentation including essential general medical information, such as premeditations, allergies, and any underlying conditions that might contribute but not be recognized by the patient who denied prior medical issues. The patient reports additional symptoms, including dark urine, oliguria, and leg swelling, as well as a history of facial swelling resulting in facial pressure and pain. Inspection reveals scratch marks consistent with nephrogenic pruritus and facial erythema consistent with cutaneous vasculitis. Small papules extend from the neck to the area behind the ears. A bloody handkerchief is visible beside the patient on the stretcher, hinting at bloody cough mucus. Upon further inspection, marked edema of the lower extremities can be noted, with visible pits remaining following manual palpation of the pretibial and ankle region.

| **Apparatus-Based Diagnostics** |  |  |
| --- | --- | --- |

Vital signs are monitored, revealing hypertension and sinus bradycardia at 40 beats per minute. Auscultation reveals fine crackles in the lungs, heart sounds are clear, rhythmic and bradycardic, and no murmurs are heard. These findings prompt further radiological evaluation as well as a 12-lead electrocardiogram (ECG). The ECG demonstrates high T waves, characteristic of hyperkalemia. A chest X-ray shows bilateral infiltrates, and participants are able to utilize virtual scrolling in a computed tomography (CT) scan of the thorax to identify typical vasculitis infiltrates in different sequences. Point-of-care ultrasound is also available, allowing participants to evaluate the size, position, and perfusion of the kidneys and rule out post-renal causes of AKI.

| **Laboratory und Urine Diagnostics** |  |  |
| --- | --- | --- |

Blood gas analysis (BGA) reveals partially compensated metabolic acidosis (pH 7.29, base excess -7.7 mmol/L, CO₂ 34.9 mmHg) and severe hyperkalemia (7.2 mmol/L). Further laboratory findings indicate elevated inflammatory markers, including a C-reactive protein level of 96 mg/L (normal level < 5mg/L), along with moderate anemia. Serum creatinine and urea levels are markedly elevated, consistent with stage 3 AKI. The autoimmune screening reveals an elevated c-ANCA titer (1:160) and the presence of PR3 antibodies. There is also an option to actively place a urinary catheter, producing a small volume of brownish, cola-colored urine suggestive of oliguria and potential blood admixtures. A subsequent urinalysis demonstrates hematuria and proteinuria through observable colorimetric changes. For further analysis, a virtual urine sediment examination is provided. Key findings include acanthocytes and erythrocyte casts. A kidney biopsy can be initiated for definitive diagnosis by requesting a nephrology consultation. The available histological images show crescent formation in the glomeruli.

| **Summary und Therapeutics Interventions** |  |  |
| --- | --- | --- |

In summary, the findings are consistent with Rapidly Progressive Glomerulonephritis (RPGN) with AKI stage 3 and, as a complication, severe hyperkalemia is present. Participants must recognize and prioritize the urgent management of life-threatening hyperkalemia (potassium level of 7.2 mmol/L) and initiate appropriate potassium-lowering treatments. These may include the administration of insulin/glucose, furosemide, cation exchangers, sodium bicarbonate, inhaled salbutamol, and calcium gluconate. Immediate feedback follows the initiation of therapy, demonstrating the resolution of bradycardia and normalization of T waves. Reassessment via blood gas analysis confirms a reduction in serum potassium levels. If RPGN caused by ANCA-associated vasculitis is suspected and correctly diagnosed, participants are expected to consider the administration of a glucocorticoid (prednisolone). Adjunctive medications, such as proton pump inhibitors, are also included to complement patient management.

Supplementary Table 2 | Design of the learning assessment questionnaire.

| **Question  (Number)** | **Question text** | **Max. points** | **Correct Answer** |
| --- | --- | --- | --- |
| Demographics Open-ended | Age:  Gender: | --  -- | --  -- |
| 1. | What type of kidney failure is indicative of a urinary transport disorder and what is the easiest way to be confirmed or ruled out? | 1 | - Postrenal kidney injury - Sonography |
| 2. | On which parameters are the different grades of severity of acute kidney injury based and how many grades exist according to KDIGO? | 3 | - Increase in creatinine concentration - Urine output over time - Three grades of severity |
| 3. | What are typical clinical features of RPGN? Name four. | 2 | - Weakness - Loss of performance - Reduce appetite - Edema - Fatigue - Hypertension - Oligo-/anuria |
| 4. | Which disorder of the acid-base balance are the following laboratory values:  pH: 7.298; pO2: 48.9 mmHg; pCO2: 34.9 mmHg; BE: -7.7 mmol/l; HCO3^-^: 17.9 mmol/l | 1 | - Respiratory partially compensated metabolic acidosis |
| 5. | Which early changes in the ECG indicates relevant hyperkalemia? | 1 | - Bradycardia - Peaked T-wave |
| 6. | Which medication do you administer to reduce hyperkalemia? Name six medications. | 6 | - Loop diuretics - Cation exchangers - Insulin/Glucose - Sodium bicarbonate - Calcium gluconate - Beta-2 sympathomimetics |
| 7. | What are abnormalities in suspected nephritic syndrome from urinalysis? | 2 | - Proteinuria - Hematuria |
| 8. | What findings in the urine sediment are indicative for glomerular damage? | 2 | - Erythrocyte cylinders - Acanthocytes |
| 9. | What are the main clinical features of acute nephritic syndrome? | 3 | - Microhematuria - Arterial hypertension - Edema |
| 10. | Which autoantibodies should be measured if RPGN is suspected? Name six of them. | 6 | - Anti-GBM antibodies - Antinuclear antibody - Perinuclear- and cytoplasmatic-antineutrophil cytoplasmatic antibody - Proteinase 3 - Myeloperoxidase |
| 11. | What are the most common types of RPGN? | 3 | - Pauci-immune glomerulonephritis/ ANCA-associated glomerulonephritis - Immunocomplex glomerulonephritis - Anti-GBM glomerulonephritis |
| 12. | What does the abbreviation “ANCA” means? | 1 | - Antineutrophil cytoplasmatic antibody |
| 13. | What are the most common organ involvements in granulomatosis with polyangiitis? | 6 | - Ear, nose and throat area - Lower respiratory tract - Skin - Myopathies and arthralgias - Kidney - Nerves |
| 14. | What diagnostics must be performed to accurately characterize glomerulonephritis? What conclusions can be drawn from this? | 4 | - Kidney biopsy - Severity - Prognosis - Therapy |
| 15. | Which histological finding is typical for highly active glomerulonephritis? | 1 | - Crescents; recent necrosis in glomeruli |
| 16. | What are the therapeutic options for the treatment of severe systemic vasculitis? Name four of them. | 4 | - Glucocorticoids - Alkylating agents (cyclophosphamide) - Rituximab - Plasmapheresis |
| 17. | Why is RPGN in particular considered a nephrological emergency that requires rapid action? Name three reasons. | 3 | - Irreversible damage to the glomeruli  🡪 sclerosis/fibrosis of the kidneys - Permanent need for dialysis - Vital threatening respiratory failure in case of pulmonary involvement |

Supplementary Table 3 | Design of the evaluation and simulation sickness questionnaire.

| **Question (Number)** | **Question type** | **Question text** |
| --- | --- | --- |
| Demographics | Open | Age:  Gender: |
| 1 | Numeric scale (1 to 10 scale) | How would you rate the general quality of the virtual reality simulation? |
| 2 | Numeric scale (1 to 10 scale) | How challenging did you found the case? |
| 3 | Numeric scale (1 to 10 scale) | How instructive did you find the simulation? |
| 4 | Numeric scale (1 to 10 scale) | In your opinion, how realistic was the simulation? |
| 5 | Numeric scale (1 to 10 scale) | How would you rate the interactivity and user-friendliness of the simulation? |
| 6 | Numeric scale (1 to 10 scale) | Did you find the feedback and learning guidance provided by the simulation helpful? |
| 7 | Numeric scale (1 to 10 scale) | How motivating did you find the simulation? |
| 8 | Numeric scale (1 to 10 scale) | How would you rate the technical implementation of the simulation? |
| 9 | Numeric scale (1 to 10 scale) | How intense was the feeling of “presence”, of actually being “on site” during the simulation? |
| 10 | Numeric scale (1 to 10 scale) | How clear was the learning objective of the simulation for you? |
| 11 | Numeric scale (1 to 10 scale) | Were you able to apply what you had learned in theory during the simulation? |
| 12 | Numeric scale (1 to 10 scale) | How did you feel about the teamwork and communication with the other seminar participants in the simulation? |
| 13 | Numeric scale (1 to 10 scale) | Did you receive sufficient feedback on your decisions during the simulation? |
| 14 | Numeric scale (1 to 10 scale) | How would you rate the graphical quality and design of the simulation? |
| 15 | Numeric scale (1 to 10 scale) | How did you feel about the audiovisual animation and quality, including background noises, voices etc.? |
| 16 | Numeric scale (1 to 10 scale) | Do you think what you have experienced and learned in the simulation could be useful in real life? |
| 17 | Open-ended | What is your overall impression of the simulation? Are there any particular aspects that struck you as particularly positive or negative? |
| 18 | Open-ended | What aspects of the simulation would you improve or change? |

| **Simulation Sickness Questionnaire** | Likert (4 point) | *For every symptom, please state in which intensity it appears in this moment:*  General discomfort  Tiredness  Headache  Straining of the eyes  Problems with sharp vision  Increased salivation  Sweating  Nausea  Difficulty concentrating  Head pressure  Blurred vision  Dizziness with eyes open  Dizziness with eyes closed  Balance problems  Stomach upset  Belching |  |
| --- | --- | --- | --- |

Supplementary Table 4 | Item-level distribution of Simulation Sickness Questionnaire (SSQ) responses after VR training. Responses were collected on a 4-point Likert scale (not at all, mild, moderate, severe). Percentages were calculated per item using the number of non-missing responses (N).

| ***SSQ item (symptom)*** | ***N*** | ***Not at all, n (%)*** | ***Mild, n (%)*** | ***Moderate, n (%)*** | ***Severe, n (%)*** |
| --- | --- | --- | --- | --- | --- |
| General discomfort | 176 | 130 (73.9) | 37 (21) | 8 (4.5) | 1 (0.6) |
| Tiredness | 180 | 151 (83.9) | 22 (12.2) | 5 (2.8) | 2 (1.1) |
| Headache | 180 | 136 (75.6) | 27 (15) | 17 (9.4) | 0 |
| Straining of the eyes | 180 | 101 (56.1) | 47 (26.1) | 25 (13.9) | 7 (3.9) |
| Problems with sharp vision | 178 | 69 (38.8) | 42 (23.6) | 39 (21.9) | 28 (15.7) |
| Increased salivation | 180 | 167 (92.8) | 10 (5.6) | 3 (1.7) | 0 |
| Sweating | 179 | 132 (73.7) | 25 (14) | 16 (8.9) | 6 (3.4) |
| Nausea | 180 | 150 (83.3) | 18 (10) | 11 (6.1) | 1 (0.6) |
| Difficulty concentrating | 179 | 133 (74.3) | 31 (17.3) | 12 (6.7) | 3 (1.7) |
| Head pressure | 179 | 128 (71.5) | 32 (17.9) | 13 (7.3) | 6 (3.4) |
| Blurred vision | 179 | 89 (49.7) | 44 (24.6) | 30 (16.8) | 16 (8.9) |
| Dizziness with eyes open | 178 | 147 (82.6) | 24 (13.5) | 5 (2.8) | 2 (1.1) |
| Dizziness with eyes closed | 178 | 156 (87.6) | 16 (9) | 4 (2.2) | 2 (1.1) |
| Balance problems | 178 | 145 (81.5) | 25 (14) | 7 (3.9) | 1 (0.6) |
| Stomach upset | 178 | 154 (86.5) | 17 (9.6) | 3 (1.7) | 4 (2.2) |
| Belching | 177 | 172 (97.2) | 4 (2.3) | 0 | 1 (0.6) |


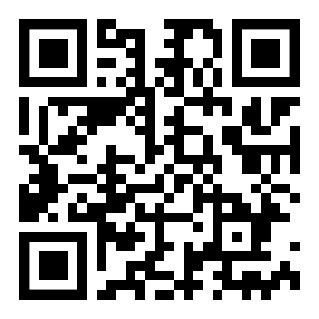

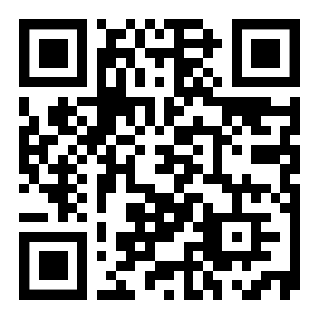

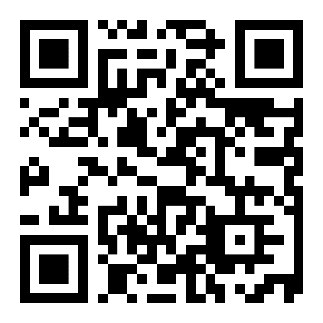


**Supplementary Figure 1. QR codes linking to exemplary first-person demonstrations of interaction within the scenario.** Scanning the QR codes provides access to short videos (urine sediment *(left)*; chest CT *(middle)*; kidney sonography *(right).*
